# Desmin interacts with STIM1 and coordinates Ca^2+^ signaling in skeletal muscle

**DOI:** 10.1172/jci.insight.143472

**Published:** 2021-09-08

**Authors:** Hengtao Zhang, Victoria Graham Bryson, Chaojian Wang, TianYu Li, Jaclyn P. Kerr, Rebecca Wilson, Deborah M. Muoio, Robert J. Bloch, Christopher Ward, Paul B. Rosenberg

**Affiliations:** 1 Department of Medicine and; 2 Duke Molecular Physiology Institute, Duke University School of Medicine, Durham, North Carolina, USA.; 3 Department of Physiology and; 4 Department of Orthopedic Surgery, University of Maryland School of Medicine, Baltimore, Maryland, USA.

**Keywords:** Muscle Biology, Calcium signaling

## Abstract

Stromal interaction molecule 1 (STIM1), the sarcoplasmic reticulum (SR) transmembrane protein, activates store-operated Ca^2+^ entry (SOCE) in skeletal muscle and, thereby, coordinates Ca^2+^ homeostasis, Ca^2+^-dependent gene expression, and contractility. STIM1 occupies space in the junctional SR membrane of the triads and the longitudinal SR at the Z-line. How STIM1 is organized and is retained in these specific subdomains of the SR is unclear. Here, we identified desmin, the major type III intermediate filament protein in muscle, as a binding partner for STIM1 based on a yeast 2-hybrid screen. Validation of the desmin-STIM1 interaction by immunoprecipitation and immunolocalization confirmed that the CC1-SOAR domains of STIM1 interact with desmin to enhance STIM1 oligomerization yet limit SOCE. Based on our studies of desmin-KO mice, we developed a model wherein desmin connected STIM1 at the Z-line in order to regulate the efficiency of Ca^2+^ refilling of the SR. Taken together, these studies showed that desmin-STIM1 assembles a cytoskeletal-SR connection that is important for Ca^2+^ signaling in skeletal muscle.

## Graphical abstract

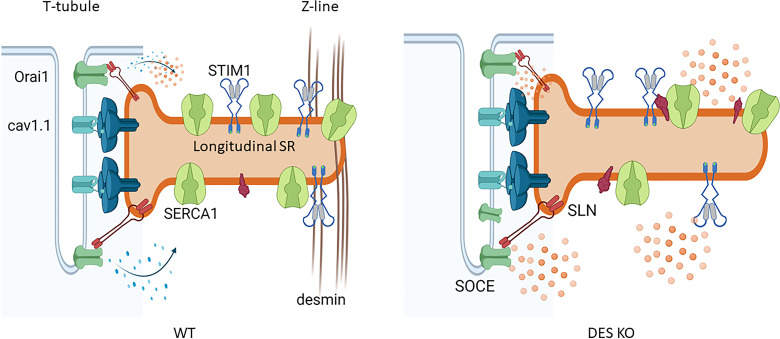

## Introduction

Stromal interaction molecule 1 (STIM1) is a multifunctional signaling protein in the sarcoplasmic reticulum/ER (SR/ER) membrane that can interact with many channels, transporters, and signaling molecules (1, 2). Understanding how STIM1-modified target proteins influence Ca^2+^ signaling in skeletal muscle remains an important yet open question. STIM1 is best recognized as an activator of Orai channels, and together STIM1 and Orai1 channels form the basic elements for store-operated Ca^2+^ entry (SOCE) (3–5). Here, STIM1 senses depletion of SR Ca^2+^ stores and activates Ca^2+^ entry through Orai1 channels. Additional STIM1 binding partners exist and include EB1, STING, POST, nuclear transporters, TRPC, PMCA, phospholamban, and SERCAs (6–10). How STIM1 regulates the function of these targets remains unclear.

Studies using mouse models and humans with STIM1 and Orai1 mutations have clarified the importance of STIM1 and SOCE in skeletal muscle development as well muscle contractility, endurance capacity, and fatigue resistance (11, 12). Reduced muscle mass and increased fatigability characterize the phenotype of mice expressing a dominant negative Orai1 channel in skeletal muscle (13) or for the selective deletion of Orai1 from skeletal muscle. Importantly, Orai1-mediated Ca^2+^ events appear to be limited to muscle development because the selective deletion of Orai1 from adult skeletal muscle results in no discernible phenotype (14). STIM1-dependent Ca^2+^ signaling, via Orai channels, regulates muscle-specific gene expression by activating calcineurin signal transduction (15–17). Mice lacking STIM1, either globally (STIM1^–/–^) or selectively in skeletal muscle (mSTIM1^–/–^), display defects in skeletal muscle growth and reduced survival (17, 18). Given this divergence in the muscle phenotype, the role of STIM1 in Ca^2+^ signaling of skeletal muscle is more complex than activation of Ca^2+^ influx via Orai1, raising the possibility of additional STIM1-targets.

In the present study, we identified desmin as an interacting partner for STIM1 from a yeast 2-hybrid screen using the cytosolic portion of STIM1 as bait. Interestingly, desmin is a type III intermediate filament protein whose expression is limited to muscle (skeletal, cardiac, and smooth). We showed that desmin interacts with STIM1 at the CC1-SOAR domain where desmin can promote STIM1 oligomerization, restrict STIM1 migration, and delay SOCE activation. We used muscle fibers from mice lacking desmin to show that the presence of STIM1 near the Z-line was diminished in isolated flexor digitorum brevis (FDB) fibers, which reduced the efficiency of Ca^2+^ refilling of the internal stores, despite augmented SOCE. Taken together, these results showed that STIM1 distributes in distinct compartments of muscle SR, including the junctional and longitudinal SR, and desmin is important for the efficiency of Ca^2+^ store refilling of the longitudinal SR.

## Results

### STIM1 resides in different domains of the SR.

**Figure 1:**
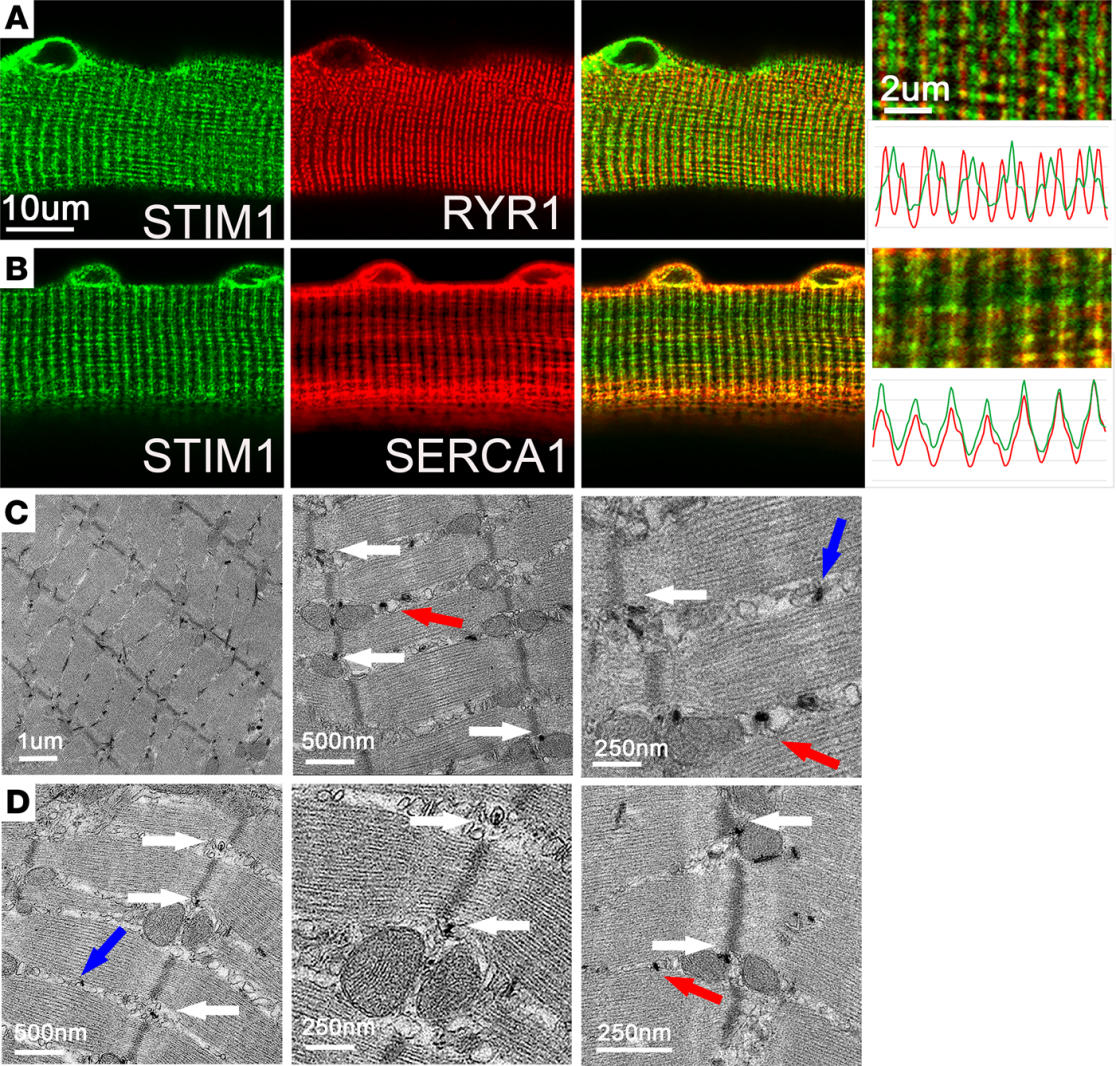
Subcellular localization of STIM1 in skeletal muscle fibers. Immunostaining was performed on isolated FDB myofibers from 6-week-old WT mice. (**A**) Representative immunofluorescence images of endogenous STIM1 alone (left), RYR1 alone (middle), and overlay (right). (**B**) Representative immunofluorescence images of endogenous STIM1 alone (left), SERCA1a alone (middle), and overlay (right). Expression profiles for markers are shown in the far-right panel. The profile color matches image color for each protein. Scale bars: 10 μm for low-magnification images and 2 μm for high-magnification images. (**C** and **D**) Distribution of STIM1-LacZ to subcellular location in muscle as determined by TEM. STIM1-LacZ expression was detected by X-gal staining of beta galactosidase activity. Blue arrows show STIM1-LacZ in longitudinal SR, red arrows show STIM1-LacZ at the triad, and white arrows show Stim1 LacZ in the SR at the Z-line. Results are representative of 3 mice.****

Immunofluorescence analysis of STIM1 localization relative to established subcellular markers was performed for isolated, fixed adult FDB skeletal muscle fibers from WT mice. STIM1 (green) colocalized partially with ryanodine receptor 1 (RYR1) (red), the principal Ca^2+^ release channel in skeletal muscle, in the terminal cisternae (Figure 1A). In addition, STIM1 (green) colocalized with SERCA1 (red), the main SR Ca^2+^ pump in muscle, which indicated that STIM1 was detected throughout the longitudinal SR along the I-band and Z-line (Figure 1B). Together these findings highlight the distribution of STIM1 throughout specialized regions of the SR, including the triadic membranes of terminal cisternae and the longitudinal SR of the I-band, Z-line region (I-Z-I).

To analyze the subcellular distribution of STIM1 in greater detail, we performed transmission electron microscopy (TEM) on skeletal muscles from heterozygous mice expressing a STIM1–β-galactosidase fusion protein (STIM1-LacZ) (17). The STIM1-LacZ fusion protein (blue) was expressed in heterozygous skeletal muscle fibers, but no staining was seen in WT fibers (Supplemental Figure 1, A and B; supplemental material available online with this article; https://doi.org/10.1172/jci.insight.143472DS1). The STIM1-LacZ fusion protein was able to insert into the SR membrane via an intact N-terminal domain and transmembrane segment, and an antibody to the N-terminus of STIM1 (green) was identical, as indicated by the line profiles (Supplemental Figure 1, C–H). In TEM micrographs of STIM1^+/lacZ^ muscle, the β-galactosidase reaction product appeared as a black precipitate, enabling us to define the subcellular localization of STIM1 in intact muscle (Figure 1, C and D). Consistent with our immunofluorescence analysis, STIM1-LacZ was localized in the longitudinal SR at Z-line (white arrows), free longitudinal SR (blue arrows), and triadic S/ER (red arrows). STIM1-LacZ in longitudinal S/ER membranes was often detected at the outer mitochondria membrane (Figure 1, C and D). Thus, STIM1 resides in different subdomains of the SR that include regions associated with Ca^2+^ entry (T-tubule) and Ca^2+^ refilling of the SR (longitudinal SR).

### Desmin interacts with STIM1 through specific C-terminal domains.

**Figure 2:**
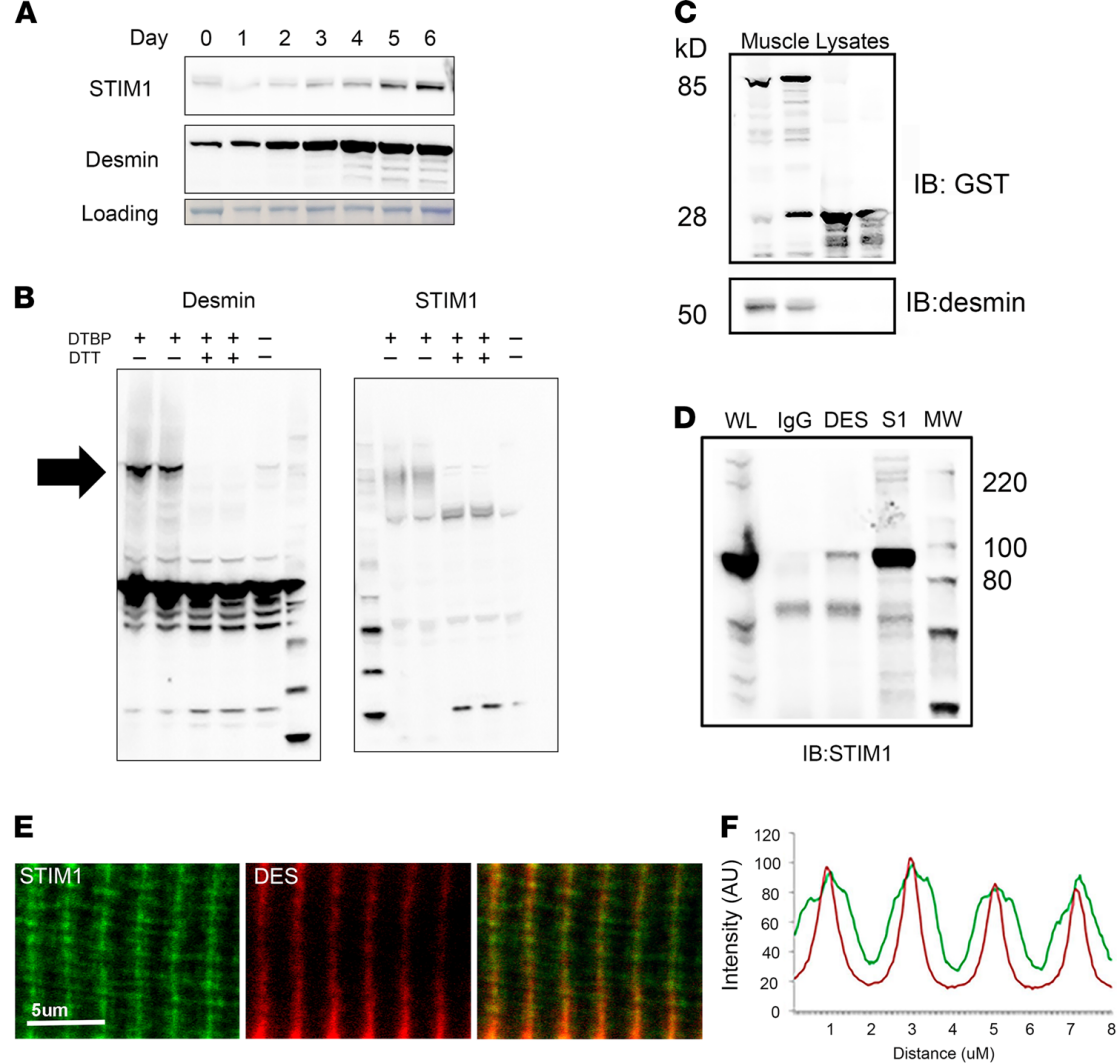
Interaction between STIM1 and desmin in the skeletal muscle. (**A**) C2C12 cells expressed STIM1 and desmin during differentiation. Expression levels of STIM1 and desmin in C2C12 cell lysates collected from day 0 to day 5 differentiation were analyzed by immunoblotting using antibodies against STIM1 and desmin, respectively. Coomassie-stained membrane was used for loading control. (**B**) Desmin and STIM1 readily cross-linked to macromolecular complexes by DTBP. Lysates from C2C12 cells were treated with DTBP or DTBP and DTT. Immunoblotting for STIM1 and desmin identified 130 kD complexes in the DTBP-treated cell lysate that were lost after DTT treatment. Arrow indicates 130 kD complex. (**C**) Verification of STIM1 and desmin interaction by GST pulldown assay. Resins that bind to GST-STIM1 were used to incubate with skeletal muscle lysates prepared from WT mice. Anti-GST antibody was used to detect fusion proteins. Desmin was detected by specific antibody only in elutes from STIM1-GST beads. Experiments were performed in triplicate. (**D**) Co-IP of endogenous STIM1 and desmin in skeletal muscle. Anti-desmin and anti-STIM1 antibodies were incubated with skeletal muscle lysate (labeled on the top). Immunoblotting with a STIM1 antibody detected endogenous STIM1 protein in the desmin IP and STIM1 IP but not the control IP with IgG. WL, whole lysate. (**E**) Immunofluorescence staining and colocalization of STIM1 and desmin from WT FDB muscle fibers. (**F**) Expression profiles for STIM1 and desmin. The profile color matches image color for each protein. Scale bars: 5 μm for high-magnification images.

To identify potentially novel muscle-specific components of the STIM1 complex, we screened a yeast 2-hybrid library generated from mouse skeletal muscle with a cytosolic portion of STIM1 (250 to 685 aa). Partial clones encoding for desmin were detected as the interacting partner for STIM1. Desmin is a type III intermediate filament protein and the most abundant intermediate filament expressed in muscle. The terminal portion of the 2B region and the tail domain of mouse desmin (394–493 aa) represent the minimal region of desmin that interacted with STIM1 in the yeast 2-hybrid assay. Desmin was expressed early in the differentiation of C2C12 myoblasts and remained stably expressed throughout this period (Figure 2A) (19). STIM1 was similarly upregulated early in differentiation, peaking after 5 days in differentiating media, which corresponded with maturation of the RYR1-containing Ca^2+^ stores (Figure 2A) (20). Both desmin and STIM1 are known to form high-ordered protein complexes (21, 22). To assess STIM1-desmin interaction, we used dimethyl dithiobispropionimidate (DTBP) to cross-link and stabilize protein-protein interactions and to determine whether desmin and STIM1 form complexes of the same size. Complexes containing desmin or STIM1 were detected in the range of 130 kD in size in the cell lysates of C2C12 myotubes (Figure 2B; black arrow identifies macromolecular complex). Specificity of the cross-linking was confirmed because addition of DTT to the lysates was able to eliminate the 130 kD complexes. During muscle differentiation, desmin and STIM1 overlapped in a temporal pattern of expression and assembled into macromolecular complexes of similar size (130 kD). While most of the desmin polymers accounted for the intermediate filament macromolecular complex, we sought to determine whether STIM1 and desmin interacted directly.

To verify the interaction between STIM1 and desmin, we used 3 independent approaches: glutathione S-transferase (GST) pulldown, co-IP, and immunostaining. First, we constructed a GST-STIM1 (251–685 aa) fusion protein in *E*. *coli* and purified it by glutathione column. Skeletal muscle lysates prepared from WT mice were applied to the column and the interaction was detected in the elute by immunoblotting for desmin. GST-STIM1 successfully pulled down endogenous desmin, but desmin did not bind to the control GST column (Figure 2C). We verified the interaction in vitro by co-IP studies. A STIM1 antibody was able to enrich endogenous STIM1 (Figure 2D, lane 4). Whereas a control IgG antibody did not precipitate STIM1, IP studies with a desmin antibody were able to precipitate STIM1 (Figure 2D). To establish whether STIM1 and desmin colocalize in muscle fibers, we used immunofluorescence with STIM1 and desmin antibodies and found that STIM1 (green) and desmin (red) at the Z-line of FDB skeletal muscle fibers (Figure 2, E and F). Collectively, these results validated our yeast 2-hybrid results and raised the possibility that intermediate filament desmin interacts with STIM1 that is located in a specific SR compartment.

**Figure 3:**
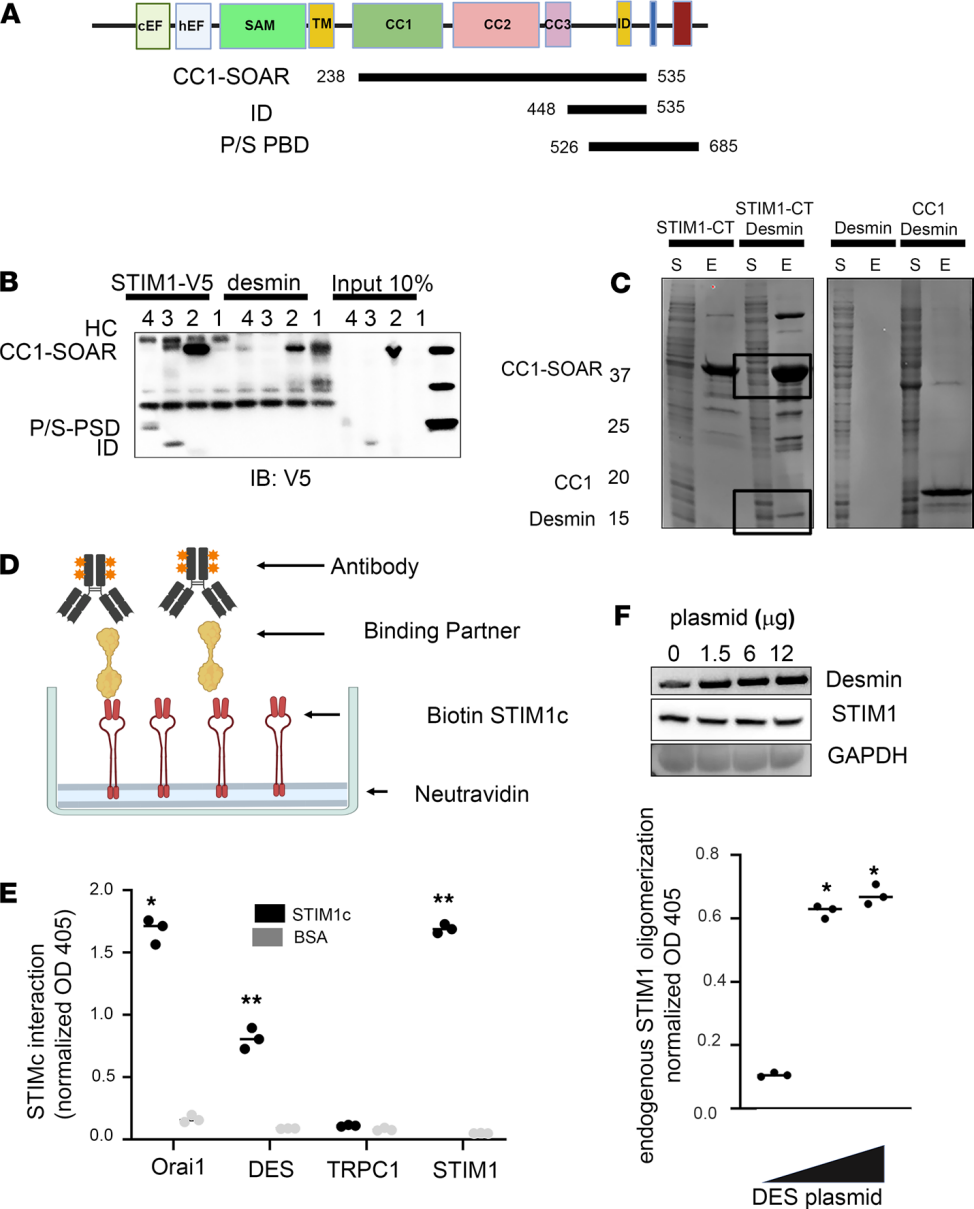
Mapping interaction of desmin and STIM1. (**A**) Diagram of STIM1 domains used for copurification with desmin. Plasmids expressing STIM1 fragments with a V5 tag were coexpressed with desmin in HEK293 cells. (**B**) Immunoprecipitates were collected with V5 or desmin antibodies. Western blotting was performed with V5 antibody to detect STIM1 fragments. CC1-SOAR is the STIM1-CT fragment. PS-PSD and inhibitory domain fragments shown in diagram were only detected in the V5 IP. (**C**) STIM1 fragments and C-terminal desmin (371–470 aa) were expressed in *E*. *coli* using pETDUET-1 to coexpress and purify proteins with cobalt beads. Coomassie-stained SDS-gels show desmin-CT fragment (11 kD) copurified with STIM1 domain CC1-SOAR (238–535 aa) (35 kD) (lanes labeled STIM1-CT desmin). Boxes identify the specific proteins. Notably, desmin fragment (371–470 aa) did not bind to beads when expressed alone (last 2 lanes). Desmin peptides did not copurify when coexpressed with STIM1-His tagged CC1 peptides (238–342 aa). S, bacterial cell lysate supernatant; E, elution of the column. (**D**) Diagram of ELISA for analyzing purified STIM1-CT bioactivity: wells were coated with NeutrAvidin and then incubated with biotin–STIM1-CT or BSA, skeletal muscle lysate, and lysate prepared from HEK293 cells that were transfected with His-tagged Orai1, which were applied to the wells, and antibodies against STIM1N-terminus, His tag, TRPC1, and desmin were used to detect the endogenous STIM1-interacting protein. (**E**) Interactions among STIM1-CT and Orai1, desmin, and TRPC1 were measured by ELISA. (**F**) Interactions between STIM1-CT and endogenous STIM1 in the presence of varying amounts of desmin were examined by ELISA. C2C12 myoblasts were transfected with desmin expression plasmid at increasing concentration. Wells coated with STIM1-CT were incubated with cell lysates from C2C12 cells expressing different levels of desmin. Endogenous STIM1 recruited to STIM1-CT was detected by anti-STIM1N antibody. OD405 absorbance was normalized by background subtraction. Values are shown as mean of triplicates ± SEM. *P = 0.00295 and **P = 0.0048 by 1-way ANOVA.

To determine the peptide domains within STIM1 that mediate the desmin interaction, we tested whether specific STIM1 fragments containing a V5 tag were able to copurify with overexpressed desmin from HEK293 cells (Figure 3A, STIM1 CC1-SOAR [251–535 aa], P/S PBD domain [526–685 aa], and the inhibitory domain [448–535 aa]). STIM1 CC1-SOAR co-immunoprecipitated with desmin, whereas other domains from the C-terminus, including the P/S domain and inhibitory domain, did not interact with the desmin (Figure 3B). To validate the CC1-SOAR domain interaction with the C-terminus of desmin, human 371–470 aa were coexpressed in *E*. *coli*. The STIM1 peptides contained a His tag, which was used to purify the CC1-SOAR domain. When expressed by itself, the STIM1-CC1-SOAR peptides appeared as a 35 kD protein in the elute. Desmin was present in the elute of the STIM1-CT pulldown as an 11 kD band, as seen in Figure 3C. Importantly, desmin-CT peptides did not bind the beads when expressed alone or when expressed with CC1-STIM1 (Figure 3C).

We next tested the hypothesis that desmin associates with the carboxy-terminus of STIM1-SOAR (STIM1-CT, 238–535 aa) in order to influence STIM1 oligomerization, a mechanism important for STIM1’s actions on Orai1. We first examined whether STIM1-CT alone was sufficient for the interaction with desmin in vitro using a cell-free assay we described previously (10). Purified biotinylated STIM1-CT protein from SF9 cells was used in an ELISA assay (Figure 3D). Lysates from skeletal muscle were added to the ELISA wells followed by antibodies to specific STIM1 binding partners, including Orai1, STIM1, and desmin. Specific antibodies detected interactions with STIM1-CT, whereas no interaction was detected in the negative controls (Figure 3, D and E). As was previously described by others, we did not detect a TRPC1 interaction with the STIM1-CT protein; this construct lacked the TRPC binding sites (Figure 3E) (23). We next used ELISA to determine whether desmin influenced the formation of STIM1-STIM1 multimers. Here, we used an antibody directed against the STIM1 N-terminus to detect endogenous STIM1. Using this STIM1 antibody, we were able to distinguish the endogenous STIM1 present in cell lysates from the STIM1-CT bound to the ELISA wells. Lysates from C2C12 myoblasts expressing increasing amounts of desmin were loaded on the ELISA plates. The binding of endogenous STIM1 to STIM1-CT was greater with increasing amounts of desmin (Figure 3F). These studies indicated that the amount of desmin present in the cell might influence oligomerization of STIM1 and likely involves the CC1-CC3 domain.

### Desmin blunts SOCE in nonexcitable cells.

**Figure 4:**
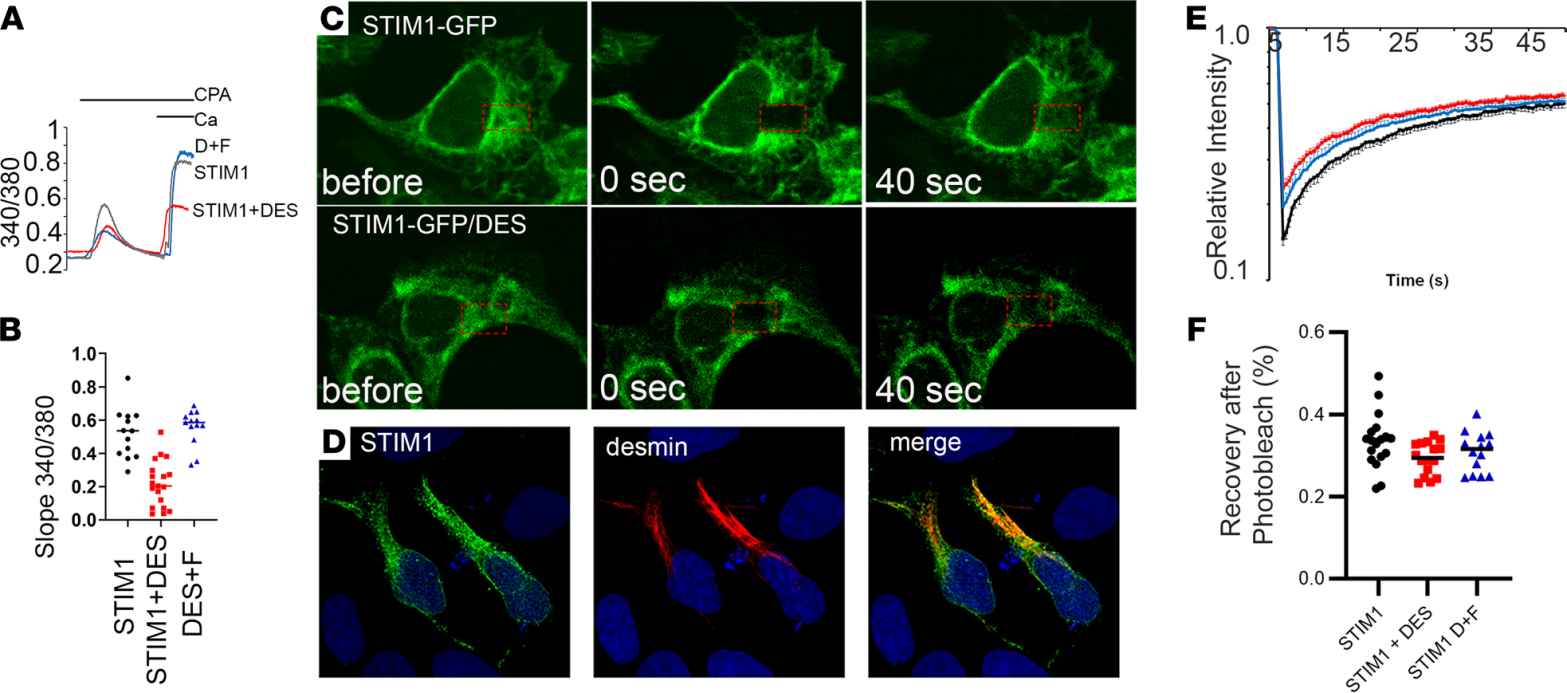
Desmin attenuates Ca^2+^ signaling in nonexcitable cells. HEK293 cells were transfected with plasmids for full-length desmin (DES) or DES and the desmin fragment (D+F; 371–470 aa). (**A**) Ca^2+^ transients were obtained by the Fura-2 method. Time course for ratio imaging is shown in **A**. Cells were perfused with a zero Ca^2+^ external solution in the presence of CPA to deplete ER stores and activate STIM1. Readdition of Ca^2+^ (10 mM) was used to assess SOCE. (**B**) Quantitative measures of SOCE as the rate of Ca^2+^ entry after Ca^2+^ was added back. Values are shown as mean ± SEM for 3 separate experiments. *P* = 0.0075 using 1-way ANOVA for the DES-expressing cells, whereas *P* = 0.0854 for D+F. *n* = 42 for WT, *n* = 55 for DES, and *n* = 14 for D+F. (**C**) FRAP experiments for STIM1-GFP. HEK293 cells were transfected with STIM1-GFP with and without desmin plasmids. STIM1-GFP alone in the top row. STIM1-GFP and desmin are present in the middle row. Time course for before and after photobleaching are present in the columns. (**D**) In the bottom panel, immunofluorescence of STIM1-GFP (left) and desmin (center) was detected in cotransfected cells. (**E**) Time course for the FRAP experiments. Rapid loss of GFP fluorescence was quantified for the different sets of cells: STIM1-GFP alone; STIM1-GFP with desmin; and STIM1-GFP with D+F. (**F**) Quantification of the FRAP recovery at 40 seconds after photobleaching for the transfected cells (*n* = 73, 97, and 81, respectively, for STIM1-GFP only, STIM1 with desmin, and STIM1 with desmin and D+F). *P* < 0.05 using 1-way ANOVA for STIM1 versus STIM1 with DES and STIM1 versus D+F. Magnification, ×60.

In cells with replete ER Ca^2+^ stores, STIM1 migrates continuously along microtubules viewed as discrete comets of ER-localized STIM1-GFP (7). Upon store depletion, STIM1 migration stops and STIM1 tubules aggregate underneath the cell membrane to activate Orai1 channels. We considered that desmin might influence these steps (22). To address this question, we used heterologous expression of full-length desmin in HEK293 cells with a standard SOCE assay and Fura-2 imaging (24). HEK293 cells were chosen because they do not express desmin as an intermediate filament, which allowed us to isolate the functional relationship of STIM1 and desmin in the regulation of SOCE. In full-length desmin–expressing cells, we noted a significant reduction in Ca^2+^ entry when Ca^2+^ was added back to store-depleted cells as reflected by a reduction in the Ca^2+^ slope and reduced SOCE amplitude (Figure 4, A and B). To validate the role of the desmin filament network in SOCE regulation, we expressed the desmin fragment corresponding to the STIM1 interacting domain, 2B coil, and C-terminal tail domain and that did not assemble into intermediate filaments. This desmin fragment restored SOCE to WT levels when expressed in DES HEK293 cells (Figure 4, A and B).

Desmin filaments might slow diffusion of STIM1-GFP in these cells and thereby limit access to Orai channels on the cell surface, resulting in limited SOCE. To test this idea, we performed fluorescence recovery after photobleaching (FRAP) experiments with STIM1-GFP. Desmin and STIM1-GFP colocalized in specific regions of the cell periphery of HEK293 cells (Figure 4D). From the confocal images, an area for STIM-GFP near the nucleus was chosen for photobleaching (Figure 4C, red dashed rectangle). Notably, recovery of STIM1-GFP fluorescence intensities after photobleaching was significantly delayed in the full-length desmin cells compared with control (CTL) cells (Figure 4, E and F). Consistent with SOCE experiments described above, the desmin interacting fragment normalized the fluorescence intensity recovery of STIM1-GFP in the desmin-expressing cells. We interpret these data as evidence that desmin intermediate filaments interact with STIM1 in the ER and delay STIM1 diffusion and thereby influence STIM1-Ca^2+^ signaling.

### STIM1 localization is altered in isolated DES-KO muscle fibers.

**Figure 5:**
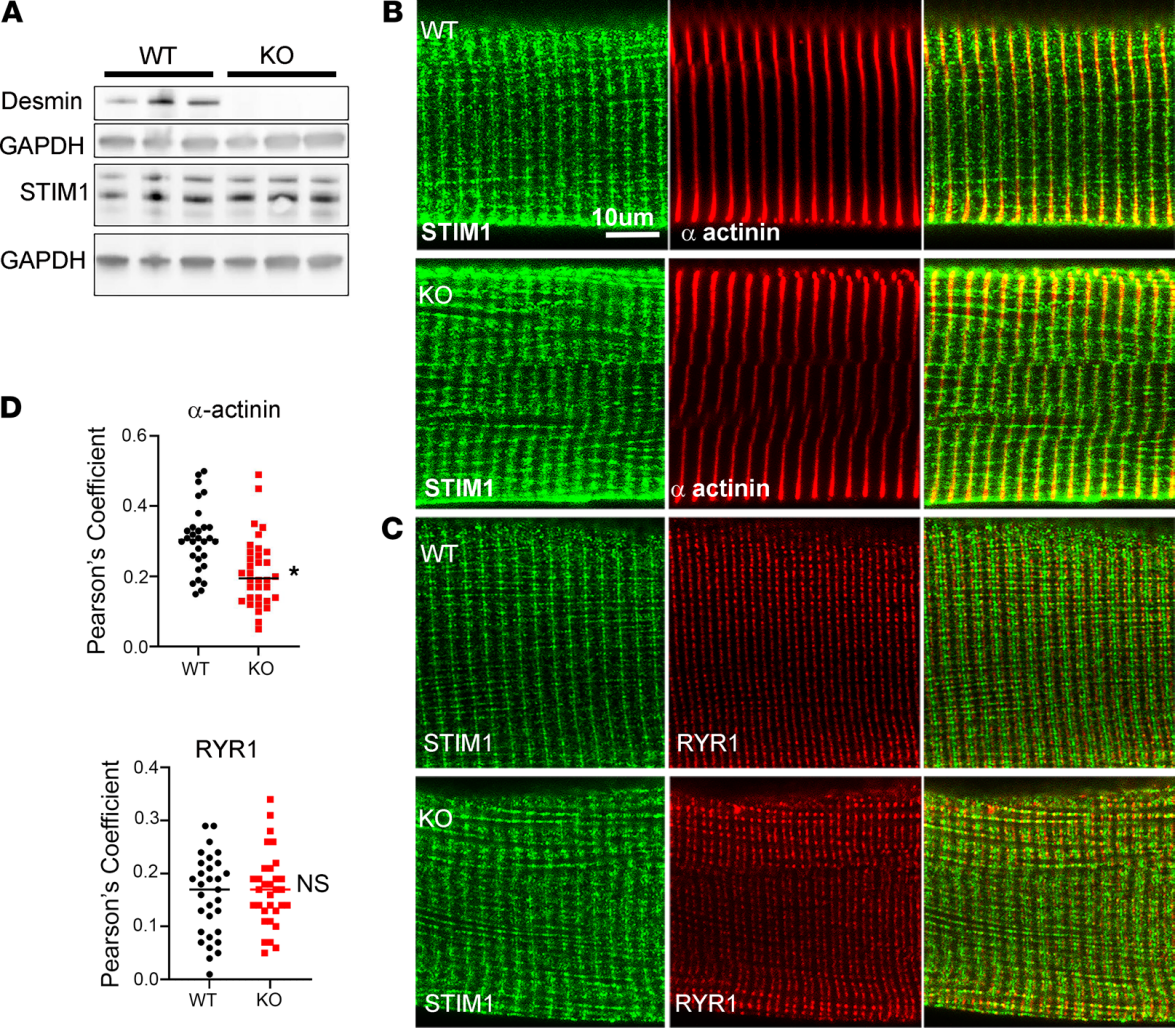
Deletion of desmin from muscle alters STIM1 localization at the Z-line. (**A**) Muscle lysates prepared from WT and DES-KO mice and Western blotting was performed with desmin and STIM1-specific antibodies. Desmin is not expressed in the DES-KO muscle. STIM1 expression is not altered in the DES-KO mice. *n* = 3 animals for each genotype. (**B**) FDB muscle fibers from WT and DES-KO mice were fixed and immunostained for α-actinin to mark the Z-lines and (**C**) RYR1 to identify junctional SR. (**D**) Colocalization studies were done for STIM1 to assess STIM1 at the Z-line and junctional SR in DES^–/–^ muscle fibers. Data were collected from 25 WT fibers (*n* = 5 mice) and 34 fibers from DES-KO mice (*n* = 5 mice). Pearson’s correlation coefficients were determined for each image. Results are shown as mean ± SEM; *P < 0.02 by 1-tailed *t* test significant only for the colocalization for STIM1 α-actinin. Scale bar: 10 μm.

To assess how the loss of desmin influenced STIM1-Ca^2+^ signaling in vivo, we turned to DES-KO mice (25). We found no significant change in STIM1 protein expression in gastrocnemius muscles of DES-KO mice (Figure 5A). However, we detected a shift in STIM1 distribution by immunofluorescence in isolated FDB muscle fibers from DES-KO mice. STIM1-specific antibodies were used to localize STIM1 by immunofluorescence with known cellular markers, including α-actinin, which marks the Z-line, and RYR1, which is a marker of the triadic SR. The STIM1 signal (green) overlapped with α-actinin (red), indicating STIM1’s presence in the longitudinal SR at the Z-line (WT; Figure 5B). Less of the STIM1 signal (green) distributed with RYR1 (red), indicating modest overlap in the terminal SR compartment, as shown previously (17) (WT; Figure 5C). We used Pearson’s correlation coefficient (PCC) as a method to compare the distribution of STIM1 with these different markers for the Z-line (α-actinin) and for the terminal SR (RYR1) for WT and DES-KO mice (26). Analysis of the PCC for STIM1-RYR1 revealed no significant difference between DES-KO and WT muscle, indicating the absence of desmin did not influence STIM1’s presence at the terminal cisternae (Figure 5, B and D). In contrast, PCC for STIM1-α-actinin showed significantly reduced colocalization in DES-KO muscle fibers compared with WT muscle fibers. This reduction in the PCC in the DES-KO fibers indicated that STIM1’s presence at the Z-line was influenced by desmin (Figure 5, C and D).

### DES-KO mice exhibit altered STIM1-Ca^2+^ signaling.

**Figure 6:**
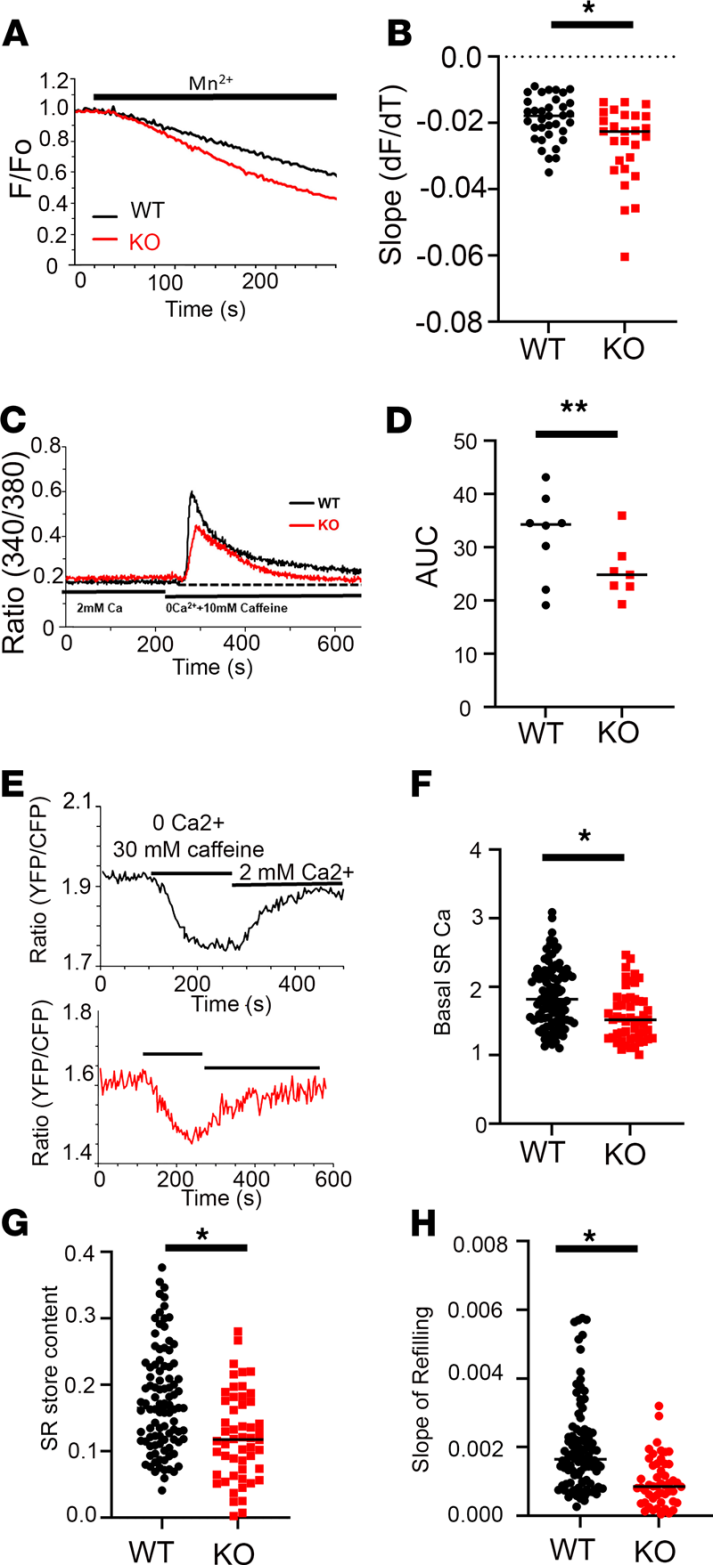
Desmin KO muscle fibers exhibit altered Ca^2+^ signaling. (**A**) Mn^2+^quench assay to compare SOCE in WT and DES-KO fibers. Fura-2 loaded WT (black) and DES-KO (red) FDB muscle fibers were imaged using 360 nm excitation, the isosbestic point for Fura-2. (**B**) The initial rates of Mn^2+^ quenching fluorescence were defined as SOCE, calculated and summarized in the Methods section. (**C** and **D**) Fura-4F loaded muscle fibers for WT (black) and DES-KO (red) subjected to SR Ca^2+^ depletion by 10 mM caffeine in the presence of 30 μM CPA. Summarized data for SR store Ca^2+^ content from WT (open column, *n* = 8) and DES-KO (filled red column, *n* = 7) FDB fibers. Significance is indicated by *P* < 0.05. (**E** and **F**) Representative FRET traces for D1ER, a genetically encoded SR Ca^2+^ indicator, electroporated into the FDB muscles. Ca^2+^ release was compared for WT (top) and DES-KO (bottom) fibers electroporated with D1ER in response to 30 mM caffeine and uptake of Ca^2+^ by SERCA. Quantification of basal Ca^2+^ level at resting state (**F**), SR Ca^2+^ content defined as Ca^2+^ release by 30 mM caffeine (**G**), and SR Ca^2+^ uptake measured as initial slope after reintroduction of 2 mM Ca^2+^ in WT (black column, *n* = 98) and DES-KO (red column, *n* = 52) FDB fibers (**H**). *P < 0.05; **P < 0.001.

To assess SOCE in the DES-KO mice, we used an Mn^2+^ quench assay for Fura-2–loaded FDB muscle fibers. The initial slope of Mn^2+^ quench of the Fura-2 signal can be used as a quantitative measure of SOCE (20). The rate of Mn^2+^ quench was much faster in the DES-KO fibers, indicating that SOCE was greater in these fibers (Figure 6, A and B). No differences in the basal Ca^2+^ levels were observed between DES-KO and FDB-WT fibers (data not shown). We next measured cytosolic Ca^2+^ transients from Fura-4F-loaded FDB fibers in the absence of external Ca^2+^ and treated with cyclopiazoic acid (CPA) to block the SERCA1 pumping and caffeine to release RYR1 stores. Here, the AUC for the Ca^2+^ release was significantly reduced in the DES-KO fibers (Figure 6, C and D). These data showed that DES-KO fibers exhibited remodeling of STIM1 Ca^2+^ signals, namely augmented SOCE and reduced SR store content. The reduction in SR Ca^2+^ release in the DES-KO fibers was investigated further using a genetically encoded Förster resonance energy transfer–based (FRET-based) indicator, D1ER, to measure free Ca^2+^ in the SR of these muscle fibers (Figure 6, E–G). We electroporated FDB muscles from WT and DES-KO mice with D1ER plasmids (27). Isolated fibers were perfused with 30 mM caffeine to empty SR Ca^2+^ stores (Figure 6E). We found that basal SR Ca^2+^, SR Ca^2+^ content, and the rate of refilling of the SR were significantly reduced in the DES-KO fibers compared with WT fibers Figure 6, E–H). We also considered that the reduced Ca^2+^ stores in the DES-KO fibers could result from excessive RYR1 leak. To assess this possibility, we carried out D1ER imaging studies with tetracaine to block RYR1 channel leak (Supplemental Figure 2A). Here, caffeine was used to deplete internal Ca^2+^ stores in the absence and presence of tetracaine (1 mM). Our results showed a small effect of tetracaine (1 mM) on the caffeine-induced store release for WT and DES-KO fibers, but this difference did not reach statistical significance. Moreover, SR Ca^2+^ stores from DES-KO were significantly reduced compared with WT fibers. (Supplemental Figure 2B).

**Figure 7:**
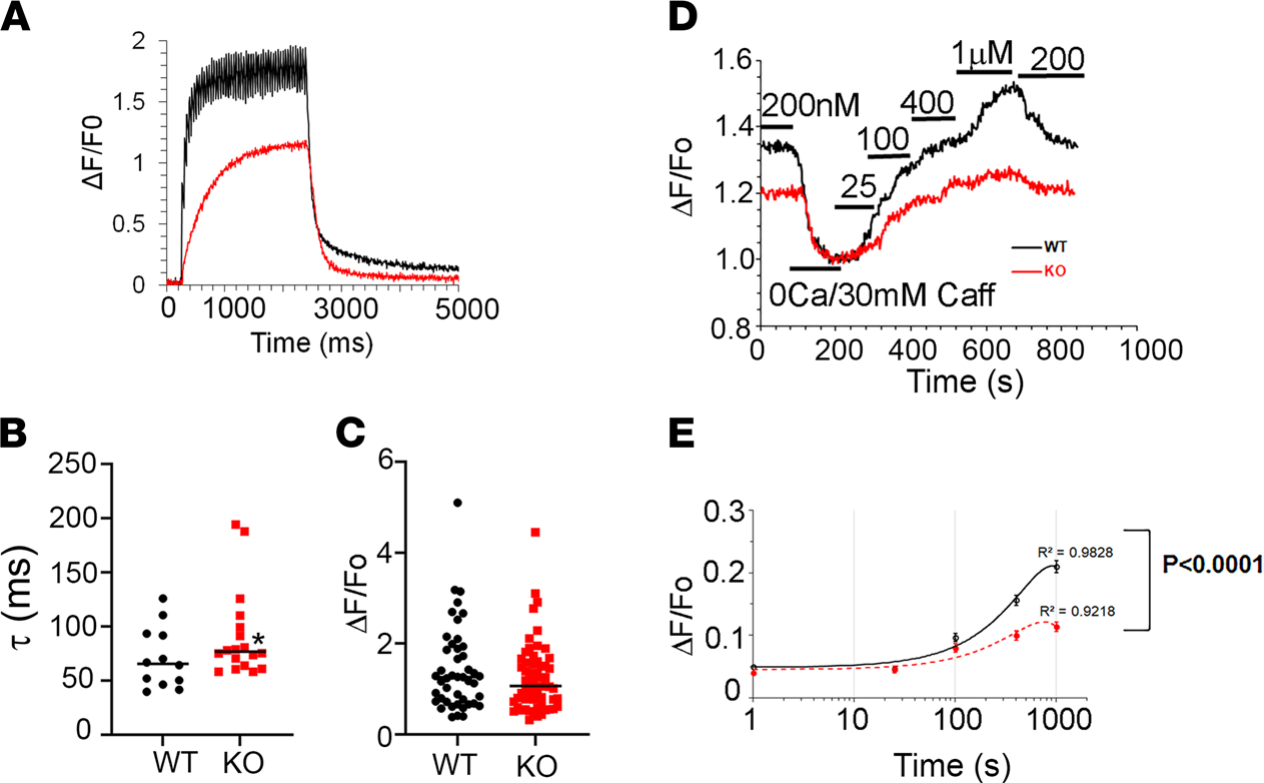
Ca^2+^ handling of SR Ca^2+^ store in the permeabilized WT and desmin KO FDB fibers and electrically evoked Ca^2+^ transients in desmin KO muscle fibers. (**A**) Flou-3 loaded FDB fibers from WT and DES-KO mice were electrically stimulated at 50 Hz for 2 seconds and Ca^2+^ transients were determined using a fast image acquisition system. The inset in **A** shows the enlarged view of decay of Ca^2+^ transients after termination of electrical stimulation. The decay time was determined for fibers after stimulation was terminated by fitting with 2 order exponentials. The decay time for DES-KO fibers (*n* = 60, *P* < 0.0001) was significantly slower than that in WT fibers (*n* = 45). (**B** and **C**) The summarized data for decay times and peak amplitude of Ca^2+^ transients from WT control and DES-KO mouse skeletal fibers after 2-second stimulation pulse. The reduction observed in DES-KO did not reach statistical significance. (**D**) Representative trace of Fluo-5N fluorescence after Ca^2+^ release from SR store by 30 mM caffeine and Ca^2+^ uptake by SR to specific [Ca^2+^]_cyto_ as indicated in the saponin-permeabilized WT and DES-KO mouse FDB muscle fibers. (**E**) Fitting curves and R^2^ of Ca^2+^ uptake by SR after Ca^2+^ release in the WT (black circle, *n* = 81) and DES-KO (red filled circles, *n* = 57) FDB muscle fibers. A significant increase (*P* < 0.0001) was noted in the WT FDB fiber at 400 nM and 1 μM [Ca^2+^]_cyto_ than in the DES-KO fibers.

Additional evidence supporting the diminished SR Ca^2+^ content in the DES-KO FDB fibers was obtained with Flou-3 imaging after electrical stimulation (50 Hz for 2 seconds) with a fast Ca^2+^ imaging system (Figure 7, A and B). The amplitude of the electrically evoked Ca^2+^ transients was reduced in DES-KO fibers, but this was not found to be statistically significant (Figure 7C). In contrast, the time interval for the decrement of the Ca^2+^ signal (tau) was significantly prolonged in the DES-KO fibers that might represent reduced refilling of the SR (Figure 7B). We noted that during 2 seconds of electrical stimulation, Ca^2+^ oscillation occurred with each stimulus in the WT FDB fibers but not in DES-KO FDB fibers.

We next designed experiments to assess SR properties in WT and DES-KO fibers when SOCE is disrupted by permeabilization of the sarcolemma and [Ca^2+^]_cyto_ is buffered by EGTA. Saponin-treated fibers were perfused with solutions that mimic cytosolic conditions and therefore clamp Ca^2+^ levels. Because fibers were loaded with the SR-Ca^2+^indicator, Fluo-5N, SR Ca^2+^ release and uptake were determined independent of SOCE. Fibers were perfused with caffeine (30 mM) and Mg^2+^ (0.01 mM) to rapidly release Ca^2+^ stores as indicated by a reduction in fluorescence intensity of the Fluo-5N signal (Figure 7D). In addition, Figure 7D illustrates the fluorescence intensity obtained from Fluo5N-loaded fibers at 4 different [Ca^2+^]_cyto_ levels. Increasing SR Ca^2+^ was detected as an increase in fluorescence intensity and reflected greater SR Ca^2+^ uptake by SERCA1. For DES-KO fibers, there was a rightward shift in the relationship between fluorescence amplitude and the [Ca^2+^], indicating diminished capacity to increase SR-Ca^2+^ (Figure 7E). These data suggest desmin has an important role in the coordination of STIM1-activated Ca^2+^ signals in skeletal muscle.

**Figure 8:**
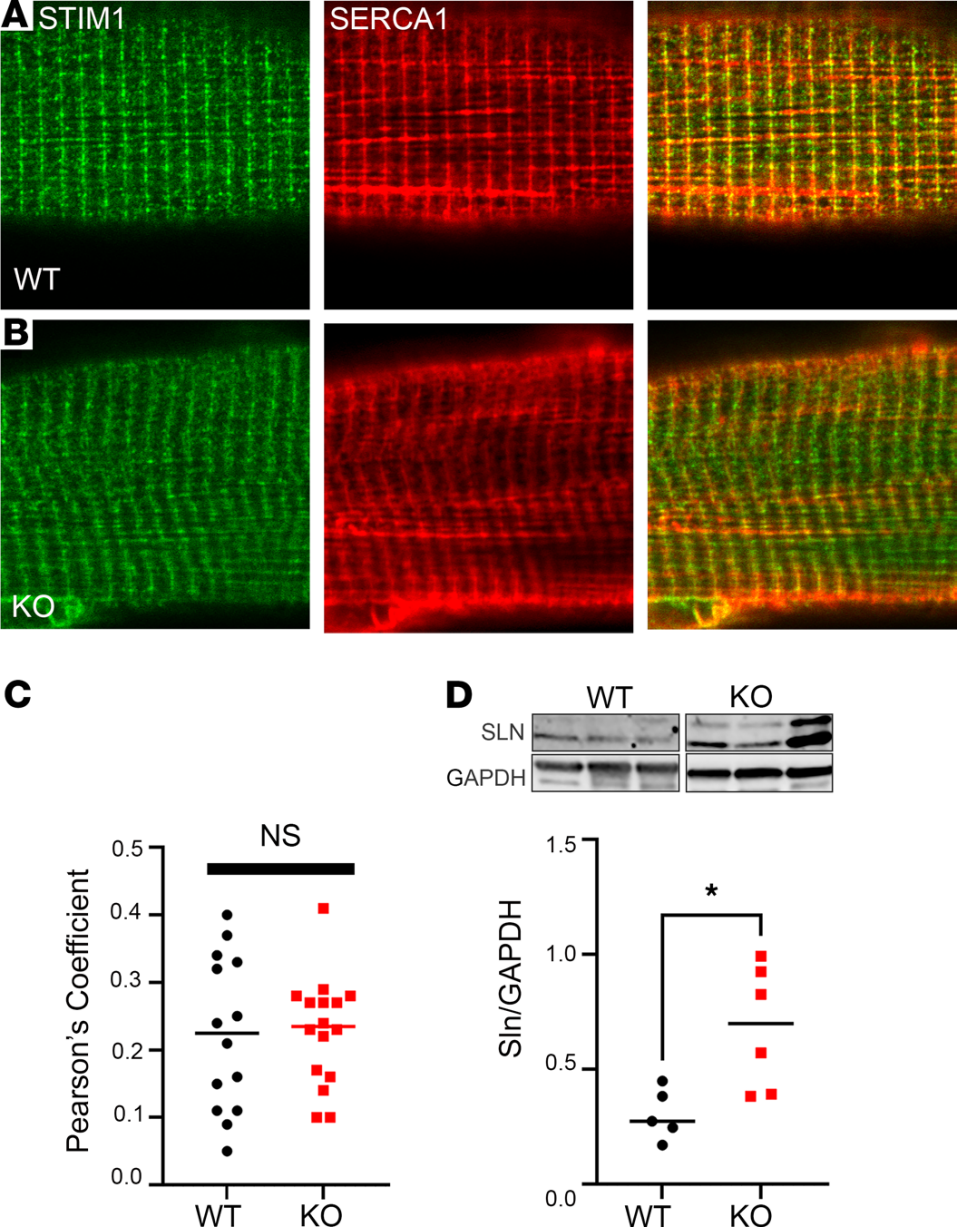
SERCA and STIM1 are displaced in the longitudinal SR at the Z-line. (**A**) Confocal micrographs of double immunolabeling for STIM1 (green, left) and SERCA1 (red, middle) was performed in (**A**) WT (top panel) and (**B**) DES-KO fibers (bottom panel). (**C**) Overlap of STIM1 (*r* = 0.225) and SERCA (*r* = 0.226) (*P* = 0.99) were quantified and compared based on the Pearson’s coefficient for WT (*n* = 3 mice, 14 fibers) and DES-KO fibers (*n* = 3 mice, 16 fibers). Results indicate overlap between STIM1 and SERCA. (**D**) Quantitative Western blotting for sarcolipin (SLN) was done for protein lysates from WT (*n* = 5 animals) and DES-KO (*n* = 5 animals) muscle. Magnification, ×63. *P < 0.05.

STIM1 and SERCA1 were both detected by immunofluorescence at the SR and I-Z-I interface (Figure 8, A and B). STIM1 expression in the DES-KO fibers (Figure 8B) was diminished at the Z-line, raising the possibility that SERCA’s presence at the Z-line might be reduced in DES-KO fibers. The PCC for STIM1 and SERCA1 overlap in WT and DES-KO fibers was not significantly different, demonstrating that STIM1 and SERCA1 localized to the same compartment of the longitudinal SR (Figure 8C). Importantly, these data suggest that displacement of STIM1 and SERCA from the Z-line in the absence of desmin may have influenced the Ca^2+^ store (Figure 6, B–D). Because our data suggest that DES-KO fibers exhibited a reduced capacity to increase [Ca^2+^]_SR_, we examined SR-Ca^2+^-handling proteins by immunoblotting. Notably, we did not find a change in the expression of the Ca^2+^ release channel (RYR1) or Ca^2+^ pump (SERCA1) levels in the DES-KO muscle (Supplemental Figure 3). We did find that sarcolipin, an endogenous inhibitor of SERCA1, was markedly upregulated in the DES-KO muscle lysate (Figure 8D). It is likely that SERCA1 and STIM1 were displaced from the Z-line of DES-KO muscles, and the upregulation of sarcolipin in DES-KO muscle contributed to disruption of the Ca^2+^ signaling in these mice. Taken together, these studies provide mechanistic insight into the reduced Ca^2+^ stores in muscle lacking desmin filaments.

## Discussion

Macromolecular complexes composed of channels, transporters, and buffers assemble to elicit Ca^2+^ transients that drive muscle contraction, gene expression, and metabolism. This is best exemplified in skeletal muscle by excitation contraction (EC) coupling, the well-established signaling complex that coordinates contraction. In addition, it has become increasingly clear that SOCE also contributes to muscle fiber Ca^2+^ dynamics and requires the assembly of a novel macromolecular complex (15, 28). It has therefore become important to define the mechanism by which STIM1 assembles into complexes with Orai1 and SERCA in skeletal muscle. Here, we identified desmin, the main type III intermediate filament in skeletal muscle, as a STIM1 binding partner. Desmin interacted with STIM1 at the Z-line of muscle fibers, whereas STIM1 was also present in the SR near the A-I band interface and the T-tubule. We found that desmin associated with STIM1 resides in the longitudinal SR to coordinate Ca^2+^ uptake to the SR, a process that is separable from STIM1-dependent activation of Orai1 in the T-tubule membrane. These findings provide mechanistic insight into the regulation of Ca^2+^ dynamics by STIM1 in muscle fibers and highlight important differences in SOCE in skeletal muscle from that described in nonexcitable cells.

Based on our immunofluorescence and TEM studies, STIM1 populated the longitudinal SR at the Z-line and the junctional SR near the triad as part of the terminal cisternae. STIM1 localized near the RYR1 channels in the triad, which is entirely consistent with local RYR1 store depletion activating STIM1 and then Orai channels at the T-tubule membrane. This SOCE is operative with each action potential of the muscle fiber, and therefore occurs on an ultra-fast time scale and does not involve STIM1 migration to form ER tubules, as occurs in nonexcitable cells (29). The growing sentiment is that this STIM1 in muscle represents the spliced variant called long STIM1 (STIM1-L), where an actin-binding domain is introduced into canonical STIM1 molecule (30). It is therefore difficult to reconcile how STIM1 at the Z-line is activating Orai1 located in the T-tubule membrane. Here, we hypothesized that Z-line STIM1 defined a second pool of STIM1. Sorting of STIM1 into these different SR domains is consistent with separate functional roles involving SOCE and SR Ca^2+^ store refilling. We focused on the STIM1 at the Z-line because desmin is present at the Z-line (31). The longitudinal SR is a specialized subdomain enriched in SERCA1 pumps and is central to the resequestration of Ca^2+^ and consequently relaxation of the fiber. How STIM1 in the longitudinal SR contributes to store refilling is likely to be distinct from how STIM1 activates Orai1. Proteomic studies have found that both STIM proteins interact with SERCA pumps in skeletal muscle as well as nonexcitable cells (1, 32, 33). In nonexcitable cells, STIM1 knockdown cells refill ER stores through microdomains involving SERCA pumps, mitochondria, and Orai1 channels (9, 34). In smooth muscle cells, overexpression of SERCA2a expands SR storage capacity, which limits SOCE and calcineurin-NFAT signaling, demonstrating that SR content and SOCE are separable events (35). STIM1 overexpression in rat cardiomyocytes increases SR Ca^2+^ store content, which involves an interaction with phospholamban (PLB), an endogenous inhibitor of SERCA2a, and likely contributes to spontaneous Ca^2+^ sparks (10). Based on observations in the present work, we believe STIM1-desmin interaction coordinates Ca^2+^ refilling of the SR and is important for the remodeling of the SR that occurs in DES-KO muscle.

The STIM1-desmin interaction may also influence the maturity and functional state of the SR. We have previously described Ca^2+^ defects in malformed DES-KO fibers that include a delay in Ca^2+^ reuptake (36). Here, we offer 2 potential mechanisms for the delayed Ca^2+^ refilling of the SR of the DES-KO fibers: an increase in sarcolipin that would inhibit SERCA1 and displacement of STIM1 and SERCA1 from the Z-line. Sarcolipin is expressed exclusively in embryonic muscle and at low levels of oxidative fibers of adult mice. Sarcolipin, by inhibiting SERCA1, limits Ca^2+^ store refilling and suppresses SOCE to prevent Ca^2+^ overload during periods of rapid muscle growth. A similar increase in sarcolipin was described in STIM1-KO neonatal muscle (24). We propose that delayed maturation of the SR in STIM1-KO mice led to diminished Ca^2+^ stores linked to sarcolipin upregulation. STIM1 and sarcolipin collaborate during muscle growth to fill newly formed stores with Ca^2+^. DES-KO muscles experience repeated bouts of postnatal degeneration and regeneration, resulting in a greater number of oxidative fibers, reduced SR Ca^2+^ stores, and disorganization of STIM1 at the Z-line (21). Tubular aggregates (TA) are another example of dysfunctional SR in skeletal muscle and develop in aging muscle and in genetic syndromes linked to mutations in STIM1 and Orai1 (37). Muscle fibers from patients with TA exhibit constitutive Ca^2+^ entry, muscle weakness, and the formation of membrane aggregates of SR. Interestingly, these tubular aggregates contain STIM1 and SERCA and form in sarcomeric regions between the I-Z-I bands of the sarcomere, which is enriched in desmin filaments (38, 39). It will be important to know whether alterations to the desmin-STIM1 complex contribute to the pathogenesis of TA myopathy.

In resting healthy skeletal muscle, STIM1 is inactive when Ca^2+^ is bound to the N-terminal luminal domain, which prevents Ca^2+^ overload and muscle damage. RYR1-Ca^2+^ release reduces luminal Ca^2+^ and therefore the amount bound to STIM1, thereby favoring STIM1 conformational changes that allow it to contact Orai1. This scenario is different from what occurs in nonexcitable cells where STIM1 moves through the cytosol attached to microtubules. Upon store depletion only, STIM1 migration stopped and assembled ER tubules adjacent to the membrane. When desmin was ectopically expressed in HEK293 cells, STIM1 was unable to efficiently activate SOCE resulting from the reduced diffusion capacity of STIM1. These data suggest that desmin serves as a break for the migrating STIM1 attached to microtubules. Additional evidence is provided that desmin strengthens the STIM1-STIM1 dimer formation, which occurs when SR Ca^2+^ stores are full (22). These studies establish a role for STIM1 at the Z-line as the STIM1-desmin interaction limits SOCE and STIM1 diffusion. Most of the STIM1 fluorescence occurs at the Z-line, so it may be that STIM1-desmin tethers STIM1 near SERCA1; all are enriched in the longitudinal SR at the Z-line (33). It is possible that STIM1-desmin facilitates the interaction of STIM1 with SERCA at the Z-line. In the present work, DES-KO fibers had less STIM1 and SERCA1 at the Z-line that correlated with reduced SR Ca^2+^ stores but accelerated SOCE. Together, the studies are consistent with a model wherein STIM1 segregates into 2 distinct populations at the T-tubule and the Z-line in order to coordinate Ca^2+^ homeostasis.

The findings we present here reconcile several recent studies regarding the mechanism of SOCE activation in skeletal muscle. Prior studies by our group showed that STIM1-KO muscle fibers fail to sustain Ca^2+^ transients during electrical field stimulation (EFS) compared with WT mice. We attributed this effect to impaired activation of SOCE and reduced SR Ca^2+^ content (17, 40). Recent studies have described a phasic SOCE that is recruited during exercise. The Launikonis group have skillfully used the skinned extensor digitorum longis (EDL) fiber model to demonstrate rapid SOCE activation with EFS (41, 42). It is apparent with this experimental preparation, SOCE is activated during each action potential (AP) and seems to require skeletal muscle training (43). Another study showed that STIM1 and Orai1 are positioned in the triadic membrane together but require store depletion to activate SOCE (13). This idea was expanded in light of findings that Orai1 in the T-tubular membrane remodels after intense exercise (44, 45). In the current study, we showed that desmin distributed STIM1 into 2 regions of the muscle to regulate SOCE and SR Ca^2+^ uptake. We used saponin-permeabilized fibers where SOCE could be separated from SERCA regulation. By assaying SR Ca^2+^ uptake in these fibers in the absence of SOCE, we showed that Ca^2+^ refilling was diminished in fibers lacking desmin. Desmin filaments are subject to calpain proteolysis during intense contractile activity, raising the possibility that disruption of the STIM1-desmin interaction might reduce STIM1 at the Z-line and limit refilling of SR Ca^2+^ stores and prompt greater SOCE (46).

In summary, we have discovered a potentially. novel interaction between STIM1 and desmin with structural and functional implications for how SOCE and SR Ca^2+^ signaling are coordinated in skeletal muscle. Importantly, we showed that the STIM1-desmin interaction modulated Ca^2+^ homeostasis in muscle by stabilizing STIM1-STIM1 dimers in the SR at regions where SERCA pumps and Orai1 channels connect to refill SR stores. The mechanistic implication of this work is that skeletal muscle can rapidly activate SOCE and refill SR Ca^2+^ stores, which involves important connections between SR membranes and the cytoskeleton. STIM1-desmin can therefore influence Ca^2+^ signaling, sustain energetic signaling, and promote muscle contractility. Disruption of the link between STIM1 and desmin may alter STIM1-Ca^2+^ signaling and contribute to the muscle degeneration and weakness associated with desminopathies. Strategies designed to regulate the desmin-STIM1 interaction may serve as an exercise mimetic or as therapy for desminopathies.

## Methods

### Animal handling

All mice were maintained in pathogen-free barrier facilities at Duke University and University of Maryland and were used in accordance with protocols approved by the Division of Laboratory Animal Resources and the IACUC at Duke University. Friend virus B (FVB) mice homozygous for the desmin^–/–^ genotype (47). STIM1^+/gt^ heterozygous mice were generated as described in Stiber et al. (17).

### Fiber isolation protocol

To isolate individual fibers, the FDB muscle was dissected from the foot carefully and immediately digested overnight at 37°C in digestion medium (DMEM with 10% FBS, 1× Pen-Strep, and collagenase A [Roche] at a concentration of 0.5–1 mg/mL). After digestion, muscles were triturated in fiber medium (DMEM with 10% FBS and 1× Pen-Strep) until individual fibers separated. Fibers were plated onto glass-bottom plates coated with 20 μg/mL laminin). Fibers were allowed a period of 2 hours to recover before experiments were performed.

### Fiber staining

Fibers were fixed in 4% PFA for 5 minutes, washed in PBS, and blocked in 10% heat-inactivated goat serum (HINGS). Fibers were incubated with primary antibody overnight at room temperature in fiber antibody solution (PBS, 2% heat-inactivated goat serum, and 0.3% Triton X-100). Primary antibodies are outlined in the Supplemental Table 1. After washing, fibers were incubated with secondary antibody for 1 hour (Molecular Probes — Alexa Fluor series) washed and mounted in VECTASHIELD. Staining was analyzed on a Zeiss 510 confocal microscope. For LacZ staining, fibers were fixed in 2% PFA with 0.2% glutaraldehyde for 10 minutes at room temperature, washed in rinse solution (5 mM EGTA, 0.01% deoxycholate, 0.02% NP40, 2 mM MgCl2), and stained in LacZ staining solution (5 mM K3Fe[CN]6, 5 mM K4Fe[CN]6, 5 mM EGTA, 0.01% deoxycholate, 0.02% NP40, 2 mM MgCl2, 1 mg mL X-gal solution) overnight at room temperature. Fibers were then washed and postfixed in 4% PFA.

### Primary antibodies

See Supplemental Table 1.

### Electron microscopy

For ultrastructural localization of STIM1-LacZ by TEM, FDB muscles were fixed in situ for 5 minutes in 2% PFA 0.2% glutaraldehyde in PBS. Fibers were then dissected and stained for LacZ as described in Stiber et al. (17). Tissue was postfixed in 2% PFA and 2.5% glutaraldehyde in 0.1 M phosphate buffer, pH 7.4, and then fixed in 1% osmium tetroxide, and stained en bloc with 1% uranyl acetate. Tissue was then dehydrated in a graded ethanol series, taken through a series of Spurr resin/ethanol washes, and embedded in Spurr resin. Thin sections were cut at 70 nm, mounted on copper grids, and counterstained with 2% uranyl acetate and lead citrate. Grids were viewed and photographed using an FEI Technai G2 Twin transmission electron microscope.

### Cell culture

HEK293 cells were cultured using standard protocols. C2C12 cells were maintained in growth media (DMEM with high glucose, 10% FBS, 100 IU/mL penicillin, and 100 Ag/mL streptomycin). To induce differentiation, the cells were switched from growth media to HEPES, insulin, and transferrin (HIT) media (DMEM high glucose with 2% horse serum, 50 mM HEPES pH 7.4, 10 μg/mL transferrin, 10 μg/mL insulin). Myotubes were used for experiments after 5 days in HIT media.

### IP, recombinant protein expression, and protein purification

Uncut gels can be viewed in the online supplemental material. Protein lysates were prepared and solubilized from gastrocnemius skeletal muscle harvested from mice using a standard RIPA buffer (50 mM Tris-HCl, pH 7.4, 1% NP40, 0.5% sodium deoxycholate, 0.1% SDS, 150 mM NaCl, 2 mM EDTA) supplemented with protease and phosphatase inhibitor cocktail. Lysates were homogenized and incubated on ice for 30 minutes. After centrifugation at 15,000*g* at 4°C, the soluble fraction was incubated with the appropriate antibodies at the specified dilution overnight. For GST pulldowns, STIM1 fragment (251–493 aa) was cloned in frame downstream of the GST epitope. GST or desmin-GST plasmid was transformed into *E*. *coli* and the GST-desmin protein was affinity-purified on the glutathione column. Muscle lysates from WT and STIM1-KO mice were prepared as above and passed through the column. After extensive washes, specific elutions were collected and subjected to SDS-PAGE. Immunoblotting with GST or STIM1 antibodies were used for detection of binding.

#### STIM1-CT and desmin coexpression and copurification in E. Coli BL21.

Desmin-CT (371–470 aa) and STIM1-CT were cloned into pETDUET-1 (Novagen). The verified plasmid was transformed into BL21 for overexpression overnight at 23°C with 1 mM IPTG induction. Cells were harvested and resuspended in binding buffer (300 mM NaCl, 20 mM Tris-Cl pH 7.5, 5% glycerol, and 5 mM imidazole and protease inhibitor from Roche). Cells were sonicated and centrifuged at 4°C, 10,000*g* for 30 minutes. Supernatant was loaded on a pre-equilibrated cobalt bead column, washed with the above buffer twice, buffered with 25 mM imidazole once, and then eluted with elution buffer (binding buffer with 300 mM imidazole). The supernatant and elution were loaded to 16% gel for detection.

#### Co-IP and Western blot.

Plasmids expressing STIM1 fragments with a V5 tag were coexpressed with desmin in HEK293 cells. V5 or desmin antibodies were incubated with cell lysate and enriched to protein A/G beads and eluted by loading buffer after sufficient washing with the same amount of IgG as the negative control. Western blotting was performed by resolving proteins at different percentage SDS-PAGE gels according to molecular weight, transferred to PVDF membrane, and then blotted with specific antibodies.

#### DTBP cross-linking.

C2C12 cells were cultured and differentiated as described above. Cell lysates were prepared in RIPA buffer in PBS (pH 7.4). Next, 100 μg total protein was used for cross-linking with DTBP (5 mM) at room temperature for 30 minutes. For a negative control, proteins were treated with DTBP (5 mM) and DTT (10 mM) at room temperature. Lysates were separated by SDS-PAGE and immunoblotted for desmin or STIM1 as described above.

### STIM1-CT protein expression and purification

cDNA sequence encoding the C-terminus of STIM1 (238–535 aa) was subcloned into the DW464 vector, which was then used to make baculovirus that expressed biotin-labeled STIM1c. Biotin-tagged STIM1c was purified using the BioBac Sf9 baculovirus purification system. Sf9 infected with STIM1c baculovirus was harvested 72 hours later by centrifugation at 448 RCF for 5 minutes (4°C). The pellet was washed in PBS and lysed in lysis buffer (50 mM Tris-HCl, pH 8.0, 10 mM β-mercaptoethanol, 100 mM KCl, 1% NP-40, 1:200 protease inhibitor cocktail III [EMD Millipore], 50 mM sodium fluoride, 50 mM β-glycerophosphate). The lysate was then loaded on to the NeutrAvidin affinity column (Pierce). The column was first washed 3 times in buffer A (20 mM Tris-HCl, pH 8.0, 500 mM KCl, 10 mM β-mercaptoethanol, 10% glycerol) and then 3 times in buffer B (20 mM Tris-HCl, pH 8.0, 1 M KCl, 10 mM β-mercaptoethanol, 10% glycerol). Purified protein was eluted from the column by elution buffer (50 mM Tris-HCl, pH 8.0, 200 mM NaCl, 2 mM EDTA, 4 mM DTT, 10% glycerol, 1 mM CHAPS, 5 mM biotin).

### ELISA

Costar 96-well plates were coated with NeutrAvidin in 100 mM NaHCO_3_, pH 8.5 overnight at 4°C. The wells were then blocked by 2% milk in 100 mM NaHCO_3_, pH 8.5 at room temperature for 1 hour. The wells were washed by PBST and 4 pmol of purified biotin-STIM1c protein was then added into each well and incubated overnight at 4°C. Negative control wells were coated with 2% milk in PBS. Skeletal muscle lysate or HEK293 cell lysate that contained overexpressed His-tagged Orai1 was incubated with the STIM1-CT–coated wells for 2 hours at room temperature and washed 5 times by PBST. Anti-desmin, anti-His tag, anti-TRPC1 (polyclonal antibody generated in our lab), and anti-STIM1 N-terminus antibodies were then incubated with the wells at the dilution of 1:1000 at room temperature for 2 hours. The wells were washed 5 times with PBST and incubated with 1:3000 secondary antibodies conjugated with HRP for 1 hour at room temperature. The wells were then washed with PBST 5 times and incubated with ABTS with 0.05%H_2_O_2_ at room temperature for 10 minutes, and the plate was read at 405 nm with a plate reader.

### Calcium imaging of FDB muscle fibers

#### Measurements of SOCE by Mn^2+^ quench and SR Ca^2+^ content.

For Mn^2+^ quench experiments, cytosolic Ca^2+^ indicator Fura-2 (2 μM) was loaded into muscle fibers plated on 35 mm MatTek dish at 37°C for 45 minutes in zero Ca^2+^ Tyrode solution including (in mM) 145 NaCl, 5.4 KCl, 0.33 NaH_2_PO_4_, 1 MgCl_2_, 5 HEPES, and 10 glucose, pH 7.4; SR Ca^2+^ was depleted with 30 μM CPA while loading dye. Next, 50 μM BTS was used to inhibit muscle contraction and reduce the motion artifact during Ca^2+^ imaging. Fura2-loaded FDB muscle fiber was then mounted to a motorized Nikon eclipse TE 2000-E microscope stage. The microscope was equipped with a Nikon 40× oil-based objective. The FDB fibers were continuously perfused with oxygenated experimental solutions. Epi-fluorescence of Fura-2 was excited by a pE-340fura illumination system (CoolLED). Ca^2+^ images were recorded with a PCO Edge 5.5 digital camera. The imaging data were acquired with MetaFluor software (Molecular Devices). After a stable baseline was recorded in the SR depletion solution, a Mn^2+^ quenching solution that contained 30 μM CPA, 2 mM Ba^2+^, and 0.5 mM Mn^2+^ was applied to activate SOCE and to quench fura-2 fluorescence. SOCE was measured as the initial slope of decrease in fura-2 fluorescence. For SR store Ca^2+^ content measurement, a low-affinity Ca^2+^ indicator, fura-4F, was used to avoid possible saturation. Next, 2 μM fura-4F was loaded in normal Tyrode solution for 45 minutes at 37°C. After 5 minutes of baseline recordings, SR store Ca^2+^ was depleted by Tyrode solution of 0 Ca^2+^, 10 mM caffeine, 10 μM verapamil, and 30 μM CPA. SR store Ca^2+^ content was calculated by integrating the area under the Ca^2+^ release curve.

### Ultra-fast Ca2+ imaging

FDB fibers were loaded with 5 μM Fluo-3 for 30 minutes in a 37°C incubator. Next, 5 mM Fluo-3 stock was reconstituted at 20% Pluronic in DMSO. Electrical field stimulation of a single FDB fiber was generated by an A310 Accupulser (World Precision Instruments) and an A385 Stimulus Isolator (World Precision Instruments) connected to the insert for a 35 mm MatTek glass-bottom dish. A 2-second duration of bursts of stimulation consisting of 1 ms current pulses (100 mA) was applied at 50 Hz. Ca^2+^ images of a single fiber were captured with a PCO Edge 5.5 digital camera at a rate up to 500 fps and acquired with micromanager software. Ca^2+^ imaging data were analyzed with ImageJ (NIH). The decay time of fluorescence immediately after a 2-second field stimulation pulse was exponentially fitted with an order of 2, defined as f(t) = A_1_e^–t/τ1^ + A_2_e^–t/τ2^ + C.

FRAP was performed with a Prairie confocal system equipped with a photoactivation/FRAP module and an Olympus BX51Wi microscope with a 60× water immersion objective. HEK293 cells transiently transfected with GF-tagged STIM1 were plated in a MatTek 35 mm glass-bottom dish. GFP fluorescence was excited with a 488 nm laser. The region of interest (ROI or mask) was defined using Prairie view software and then photobleached with a 405 nm laser for 50 ms at maximal laser power. Fluorescence recovery of ROI after photobleaching was recorded continuously for 50 seconds. The intensities of fluorescence in the photobleached area were analyzed and normalized to that before photobleaching.

### D1ER measurements of SR Ca2+ content

The plasmid encoding the D1ER SR Ca^2+^ indicator was electroporated into FDB fibers of 2-month-old FVB (CTL) and DES-KO mice. Then, 10 to 14 days after electroporation, single FDB muscle fibers were isolated using the enzyme digestion method. Fibers were plated to a glass-bottom 35 mm MatTek dish and mounted to a Nikon TE2000-E microscope for Ca^2+^ imaging. The D1ER was excited with a 436 nm LED light (CoolLED). Cyano and yellow fluorescent proteins (CFP and YFP, respectively) fluorescence were filtered with narrow-band FRET filters installed inside a motorized cubic filter wheel and collected with a 250 ms delay. Ca^2+^ imaging data were recorded and analyzed with MetaFluor software (Molecular Devices). SR Ca^2+^ was released using a 0 mM Ca^2+^ Tyrode solution containing 30 mM caffeine. The contraction of muscle fiber was inhibited with myosin inhibitor BTS (50 μM).

### Fluo-5N measurement of Ca2+ uptake to the SR

To assay SR Ca^2+^ with Fluo-5N fluorescence, as previously described (48), isolated FDB fibers were loaded with 5 μM Fluo 5N at 37°C in an incubator for 2 hours followed by a 15-minute de-esterized step in normal Tyrode solution with 50 μM BTS. Fluo-5N–loaded fiber was then permeabilized with 0.01% saponin in a relaxing solution including (in mM) 120 K-glutamate, 50 Trizma maleate, 1 EGTA, and 2 MgCl_2_ for 90 seconds. Permeabilized muscle fibers were then bathed in a standard internal solution containing (mM) 50 EGTA, 36 NaCl, 10 HEPES; 126 K^+^, 8 ATP, 10 creatine phosphate, and 1 Mg^2+^, pH 7.2. Cytosolic free Ca^2+^ concentration was calculated with a web-based program (http://somapp.ucdmc.ucdavis.edu/pharmacology/bers/maxchelator/webmax.htm). Next, 0–42 mM CaCl_2_ was added to standard internal solution as previously described (49). Ca^2+^ was released from SR using a standard internal solution that contained 0 mM Ca^2+^, 0.01 mM Mg^2+^, and 30 mM caffeine. Fluo-5N was excited by a 470 nm LED light (CoolLED pE 340). Emission of fluorescence was collected and recorded with a PCO Edge camera controlled by MetaFluor software (Molecular Devices). Ca^2+^ imaging data were further analyzed after subtraction of background fluorescence and normalized with F/F_0_.

### Statistics

Averaged results are presented as mean ± SEM. Based on a power calculation using 95% CI and assuming a 20% difference in the magnitude of the effect between groups, a sample size of *n* (mouse and muscle tissue) for each group was between 8 and 20 to achieve a statistical power of 80% (beta = 20%). Fibers (WT vs. KO) were allocated randomly after isolation for Ca imaging and histology; otherwise, no randomization was performed. The principal operator was blinded to the genotypes for calcium imaging and histology. Comparisons were made among gender- and age-matched controls for several independent litters. No data were excluded from analysis. All data points represent biological replicates. Population means were tested for significance using a Student’s 2-tailed *t* test. A *P* value less than 0.05 was considered significant.

## Author contributions

Yeast-2 hybrid was performed by PBR. TL, JPK, and C Wang conducted the STIM1 biochemistry and molecular studies. Mice were bred and maintained by VGB, RW, and HZ. Immunofluorescence was performed by VGB and JPK. TEM studies were performed by VGB. Calcium imaging was performed by HZ and C Ward. PBR and C Ward interpreted the data and wrote the manuscript. DMM and RJB contributed to interpretation and writing of manuscript. All authors contributed to editing of the manuscript and figures.

## Supplementary Material

Supplemental data

## Version 1 09/08/2021

Electronic publication
