# Supplementary material for: Desmin interacts with STIM1 and coordinates Ca2+ signaling in skeletal muscle
Source: JCI Insight. 2021 Sep 8;6(17):e143472. doi: 10.1172/jci.insight.143472 (PMC8492340; doi:10.1172/jci.insight.143472)
Supplement: Supplemental data [file jciinsight-6-143472-s240.pdf]

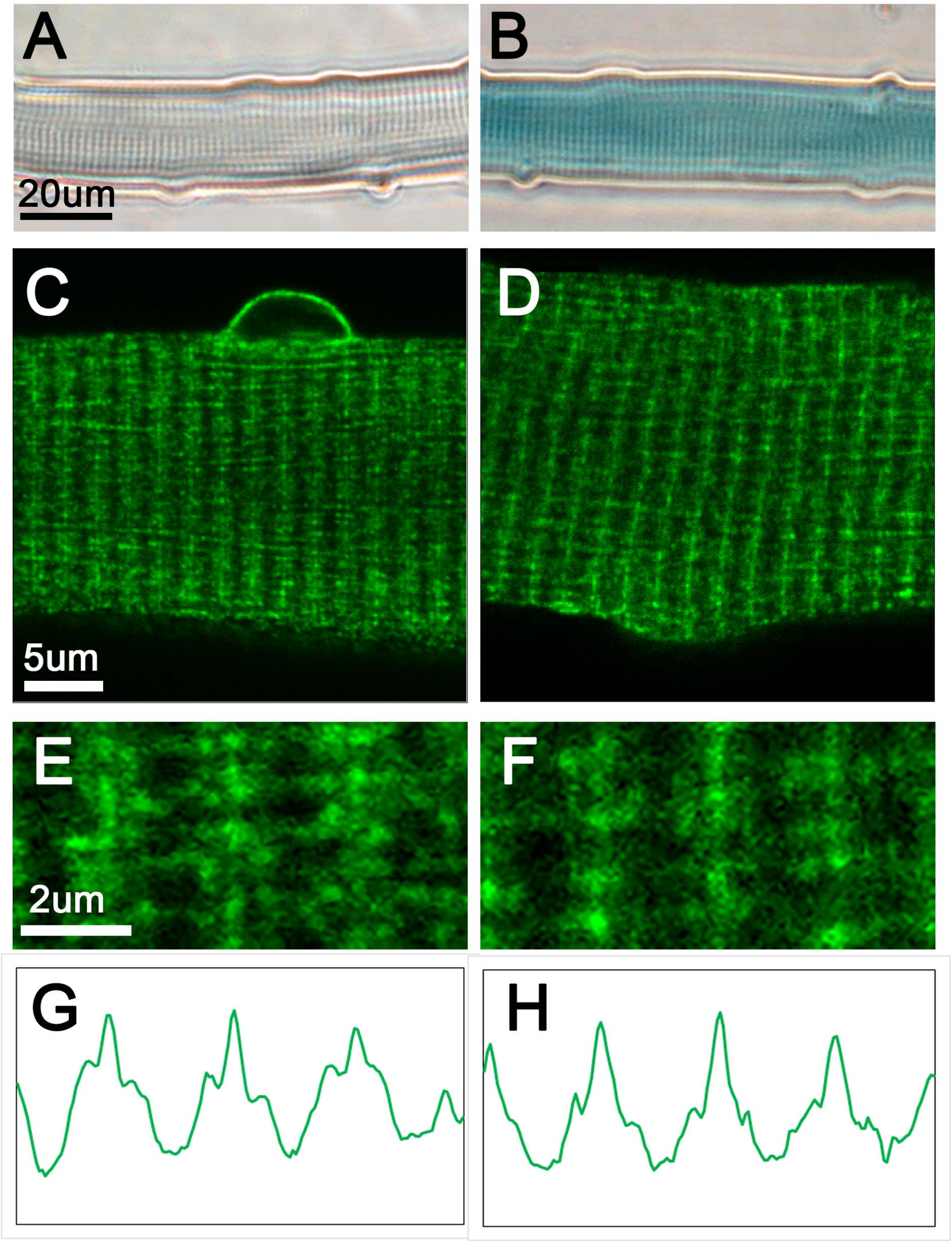

Supplemental Figure 1: Stim1 and STIM1-LacZ localization in WT and STIM1$^{+/gt}$ FDB fibers. **A)** FDB fibers after x-gal staining reveals Stim1-LacZ localization (left, blue precipitate) as demonstrated by x-gal staining of STIM1$^{+/gt}$ fibers. **B**) No staining is detected in the WT fibers

(right) **C-F)** Antibody staining of Stim1 (green) in WT (C and E) and STIM1$^{+/gt}$ mice (D and F). **G-H)** Profiles of expression data shown in images E) WT and F) STIM1$^{+/gt}$ respectively.

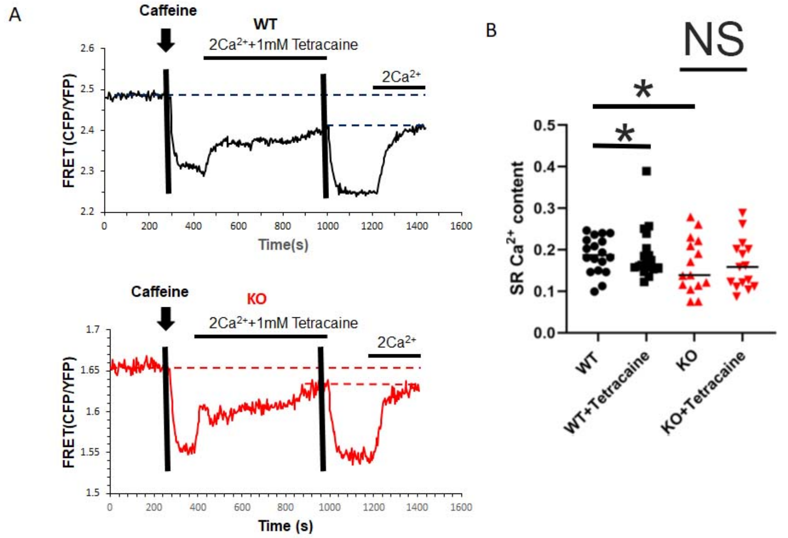

Supplemental Figure 2. The effect of tetracaine on SR Ca^{2+} content and uptake in WT and Des KO mouse FDB fibers. A, representative traces of SR Ca2+ release from WT and Des KO FDB fibers in the presence of 1 mM tetracaine and in the absence of tetracaine. Ca^{2+} from SR was released by 30mM caffeine as indicated by a vertical black bar and arrow. B: summarized data of SR Ca^{2+} release by caffeine in the presence and absence of 1mM tetracaine from WT (n=18) and Des KO (n=15) FDB fibers.

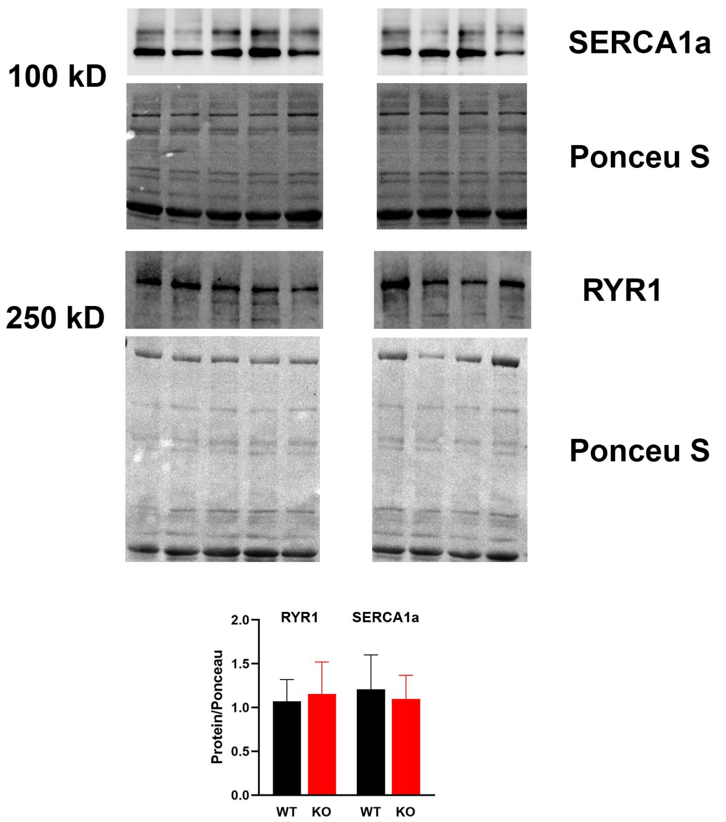

Supplementary Figure 3: Ca^{2+} handling proteins in the WT and DES KO muscle. A) Confocal micrographs of double immunolabeling for STIM1 (green, left) and SERCA1 (red, middle) was performed in WT (top panel) and DES KO fibers (bottom panel). Overlap of STIM1 ($r=0.225$) and SERCA ($r=0.226$) ($p=0.99$) were quantified and compared based on the Pearson's coefficient for WT (n=3 mice, 14 fibers) and DES KO fibers (n=3 mice, 16 fibers). B) Western blot of RYR1 and SERCA in 5 preparations from WT and DES KO muscle. GAPDH was blotted from the same membrane for a loading control. C) Quantification of normalized protein level of SERCA1 in WT (n=5) and DES KO (n=5) muscle fibers, no significant difference between two groups was found. D) Quantitative of RT-PCR with primers specific for sarcolipin (SLN) in the WT (n=5) and DES KO (n=5) muscle fibers.

Supplementary Table: Antibodies used for studies in manuscript.

Antigen	Host	Dilution	Manufacturer	Cat #	Use
Stim1 C terminus	Mouse mono	1:2000	Affinity Bio	MA1--19451	WB, IP
Stim1 N terminus	Mouse mono	1:200	BD Bioscience	610954	IF
Stim1 N terminus	Rabbit poly	1:2000	Protein Tec	11565-1-AP	WB, ELISA
Orai1	Rabbit poly	1:100	Affinity Bio	Specially made	IF
Orai1	Rabbit poly	1:2000	Alamone	ACC-062	WB
Desmin	Rabbit poly	1:50	Abcam	Ab15200	IF
Desmin	Rabbit poly	1:2000	Abcam	Ab8592	WB, IP
Poly histidine	Mouse mono	1:3000	Sigma	A1029	IF, ELISA
Serca1	Mouse mono	1:1000	Thermo Fisher	VE121G9	Wb, IP
TRPC1	Rabbit poly	1:1000	Affinity Bio	Specially made	ELISA
SC71 - Myosin 2a	Mouse	1/100	Hybridoma	SC71c	IF
BF-F3 – Myosin 2b	Mouse	1/100	Hybridoma	BF-F3c	IF
Mouse IgM 488	Goat poly	1/500	Molecular probes		IF
Mouse IgG ɣ1 555	Goat poly	1/500	Molecular probes	A21127	IF
Mouse IgG 488	Goat poly	1/500	Molecular probes	A11029	IF
Rabbit 488	Goat poly	1/500	Molecular probes		IF
Rabbit 555	Goat poly	1/500	Molecular probes		IF
